# Protocol: rhPCR and SNaPshot assays to distinguish *Plasmodiophora brassicae* pathotype clusters

**DOI:** 10.1186/s13007-022-00923-w

**Published:** 2022-07-02

**Authors:** Heather H. Tso, Leonardo Galindo-González, Troy Locke, Stephen E. Strelkov

**Affiliations:** 1 Department of Agricultural, Food and Nutritional Science, University of Alberta, Edmonton, AB T6G 2P5 Canada; 2 Present Address: Ottawa Plant Laboratory, Science Branch, Canadian Food Inspection Agency, 3851 Fallowfield Road, Ottawa, ON K2H 8P9 Canada; 3 Department of Biological Sciences, University of Alberta, Edmonton, AB T6G 2E9 Canada

**Keywords:** *Brassica napus*, Clubroot, Diagnostics, Detection, *Plasmodiophora brassicae*, Pathotyping, PCR, rhPCR, SNaPshot, Sequencing

## Abstract

**Background:**

Clubroot of canola (*Brassica napus*), caused by the soilborne pathogen *Plasmodiophora brassicae*, has become a serious threat to canola production in Canada. The deployment of clubroot-resistant (CR) cultivars is the most commonly used management strategy; however, the widespread cultivation of CR canola has resulted in the emergence of new pathotypes of *P. brassicae* capable of overcoming resistance. Several host differential sets have been reported for pathotype identification, but such testing is time-consuming, labor-intensive, and based on phenotypic classifications. The development of rapid and objective methods that allow for efficient, cost-effective and convenient pathotyping would enable testing of a much larger number of samples in shorter times. The aim of this study was to develop two pathotyping assays, an RNase H2-dependent PCR (rhPCR) assay and a SNaPshot assay, which could quickly differentiate *P. brassicae* pathotypes.

**Results:**

Both assays clearly distinguished between pathotype clusters in a collection of 38 single-spore isolates of *P. brassicae*. Additional isolates pathotyped from clubbed roots and samples from blind testing also were correctly clustered. The rhPCR assay generated clearly differentiating electrophoretic bands without non-specific amplification. The SNaPshot assay was able to detect down to a 10% relative allelic proportion in a 10:90 template mixture with both single-spore isolates and field isolates when evaluated in a relative abundance test.

**Conclusions:**

This study describes the development of two rapid and sensitive technologies for *P. brassicae* pathotyping. The high-throughput potential and accuracy of both assays makes them promising as SNP-based pathotype identification tools for clubroot diagnostics. rhPCR is a highly sensitive approach that can be optimized into a quantitative assay, while the main advantages of SNaPshot are its ability to multiplex samples and alleles in a single reaction and the detection of up to four allelic variants per target site.

## Background

The obligate parasite *Plasmodiophora brassicae* Woronin is the causal agent of clubroot, an important soilborne disease of crucifers worldwide. In Canada*, P. brassicae* is a major constraint in the production of canola (*Brassica napus* L.), with the disease managed primarily by planting clubroot-resistant (CR) cultivars. However, the overuse of CR cultivars has resulted in shifts in the virulence of *P. brassicae* populations and the proliferation of novel pathotypes that can overcome genetic resistance. Resistance-breaking pathotypes of the clubroot pathogen were first identified in 2013 [1], just 4 years after the introduction of CR canola to the Canadian market. Since then, novel virulent pathotypes have been identified every year from infected canola plants [2–5].

Traditionally, the classification of *P. brassicae* into pathotypes has relied on bioassays conducted with host differential sets. In brief, isolates of *P. brassicae* are inoculated onto a series of differential hosts, and then grouped into pathotypes based on their virulence patterns on these hosts. Various differential systems have been developed, including the hosts of Williams [6], Somé et al. [7], the European Clubroot Differential (ECD) set [8] and, most recently, the Canadian Clubroot Differential (CCD) set [4] and Sinitic Clubroot Differential set [9]. The CCD set is now the most widely used differential system in Canada, and was developed to improve identification of resistance-breaking pathotypes recovered from canola [2, 4]. While effective, the use of any host differential set is time-consuming, labor-intensive, and requires biosecure plant growth facilities. This can limit the number of isolates that can be tested, as well as the speed at which results can be obtained. Environmental factors and the specific growing conditions may also influence host reactions, thereby potentially reducing the consistency of results. The ability to detect pathotypes quickly and efficiently has become a priority for clubroot management efforts, especially with the rapid emergence of new pathotypes capable of overcoming the resistance in many CR canola cultivars. Molecular assays would facilitate rapid pathotype identification and enable prompt testing of much larger numbers of samples.

Various molecular markers have been explored for *P. brassicae* pathotyping. A random amplified polymorphic DNA marker specific to pathotype P_1_, as defined on the differentials of Somé et al. [7], was identified and converted into a sequence-characterized amplified region [10]. The *Cr811* gene, which was hypothesized to have a role in clubroot pathogenesis, was found to be exclusive to pathotype 5 [11], as defined on the differentials of Williams [6], and hence could serve a diagnostic purpose. A region of the 18S internal transcribed spacer sequence specific to pathotype 5X, as defined on the CCD set, was used to develop a probe-based qPCR assay for the specific detection of this pathotype [12]. Five molecular markers were found to distinguish pathotypes 4, 7, 9, and 11 [13], as classified on the differentials of Williams [6]. Recently, over 1500 single nucleotide polymorphisms (SNP) were identified as differentiating two genetically distinct *P. brassicae* populations from Alberta, Canada, enabling the development of population-specific molecular markers [14, 15]. Two RNase H2-dependent PCR (rhPCR) [16] primer pairs were also developed to differentiate a new, resistance-breaking “pathotype 3-like strain” of *P. brassicae* from the original pathotype 3 [17]. However, neither the exact nature of this pathotype 3-like strain, nor its CCD classification, were available. To our knowledge, no rhPCR-based assays have been reported to distinguish between pathotype clusters in *P. brassicae* isolate collections. Similarly, there are no reports of the use of SNaPshot technology [18] for the identification of *P. brassicae* pathotypes.

The novel allelic discrimination technology, rhPCR, provides greater accuracy and sensitivity relative to conventional PCR [16]. In conventional PCR, the differentiation of pathotypes with slight nucleotide variations is challenging, since non-targets may also be amplified. The rhPCR primers are blocked by a single ribonucleotide residue positioned at the discriminatory SNP, and a 3′ C3 spacer that prevents polymerase extension activity. Amplification with rhPCR requires perfect binding of primers to the target, allowing differentiation of samples with a single nucleotide difference. The blocked primers are activated via cleavage of the ribonucleotide by the RNase H2 enzyme from *Pyrococcus abyssi*, which removes both the ribonucleotide and the C3 spacer. The enzyme can only unblock the primer if the ribonucleotide is complementary to the template strand as the enzyme binds to RNA-DNA duplexes. In the case of a mismatch, no cleavage will occur due to a bulge at the mismatch site, causing steric hindrance of the enzyme, thus the primer remains blocked, and no amplification occurs during PCR.

SNaPshot is a modified sequencing single base extension reaction that enables discrimination based on SNPs [18]. Differentiating SNPs are identified based on a fluorescent color corresponding to one of the four possible alleles. SNaPshot primers are positioned one base upstream of the SNP. When the primer anneals to the DNA template, the polymerase extends the primer by one base with a fluorescently labelled dideoxynucleotide (ddNTP) matching the SNP. The resulting product size is the length of the SNaPshot primer plus the additional ddNTP that stops further extension of the product. The SNaPshot product is then analyzed via capillary electrophoresis, and pathotypes are identified based on the color of the fluorescence and product size. The signal will consist of one fluorescent color if a single allele is present, or two or more colors if multiple allelic variants are present in the same target site. Multiple targets in the genome can be assessed concurrently if necessary by varying the length of primers.

Here, we report and validate two independent assays based on rhPCR and SNaPshot technologies to differentiate between a pathotype 5X cluster and a pathotype 3H cluster of *P. brassicae,* as defined on the CCD set. Clusters are formed among several pathotypes, based on the allelic variant in the targeted discriminatory SNP positions used as molecular markers for assay development. Additional pathotypes, clustering with either pathotype 5X or 3H based on the allelic variant in the discriminatory SNP positions, were also tested. Pathotype 5X was selected for this study as it is the first and best characterized of the pathotypes able to overcome the resistance in CR canola, while pathotype 3H was included as it is one of the most common pathotypes from canola in western Canada and cannot overcome host resistance [3, 4]. Our results suggest that rhPCR and SNaPshot technologies can provide a simple and reliable way to distinguish pathotypes of *P. brassicae* in a rapid manner.

## Results

### RNase H2-dependent PCR

**Fig. 1:**
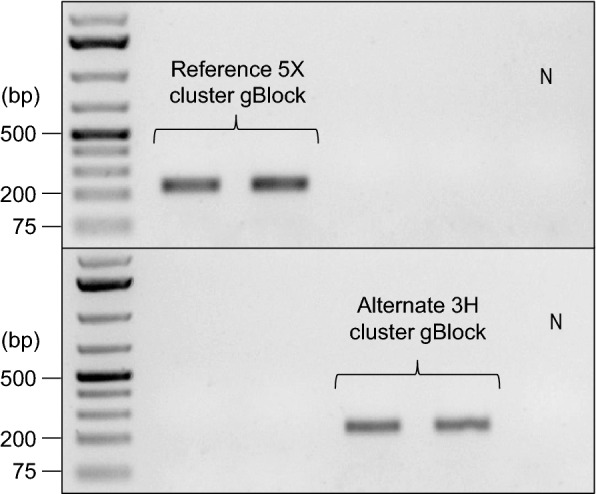
Testing of the specificity of the rhPCR assay with gBlocks. Amplicons were resolved by electrophoresis on 1% (w/v) agarose gels prepared with Tris-acetate-EDTA buffer and SYBR Safe gel stain. A GeneRuler 1 kb Plus DNA Ladder (Thermo Scientific, Waltham, MA, USA) was included in the leftmost lane as the marker. The primer pairs rh1-43812R (upper panel) and rh1-43812A (bottom panel) were evaluated against the gBlocks. Two replicates of the reference 5X cluster gBlock and two replicates of the alternate 3H cluster gBlock were ran with each primer pair. The negative control is displayed in the rightmost lane, as represented by the N

We designed two sets of rhPCR primer pairs to distinguish *P. brassicae* isolates belonging to either one of the pathotype clusters (Table 1). The specificity of the primers and the rhPCR block-cleavable technology was tested with gBlocks gene fragments. The amplification of the gBlocks with rhPCR generated bands of the expected 230 base pair amplicon (Fig. 1). The primer pair rh1-43812R was specific to the gBlock designed to replicate the reference polymorphic sequence, and yielded no visible PCR products with the alternate gBlock. The primer pair rh1-43812A was specific to the gBlock designed to replicate the alternate polymorphic sequence, and yielded no visible PCR products with the reference gBlock. This confirmed that the rhPCR primer pairs were specific to the targeted polymorphic sequences. No amplification occurred with the no RNase H2 enzyme control (results not shown).

**Table 1:** The rhPCR primer sequences designed to cluster *Plasmodiophora brassicae* pathotype DNA

Primer pair	Primer name	Primer sequence (5′-3′)^c^
rh1-43812R^a^	rh1-43812Rfw	**A**CGACG**A**CCCGGACAC**C**ATCG**CrU**AACGC/3SpC3/
	rh1-43812Rrv	TTGGCGATGG**G**CGCCACC**rU**GCGAT/3SpC3/
rh1-43812A^b^	rh1-43812Afw	**G**CGACG**T**CCCGGACAC**T**ATCG**TrC**AACGC/3SpC3/
	rh1-43812Arv	TTGGCGATGG**T**CGCCACC**rG**GCGAT/3SpC3/

^a^Primer pair to amplify pathotypes of the reference cluster (pathotype 5X)

^b^Primer pair to amplify pathotypes of the alternate cluster (pathotype 3H)

^c^The discriminatory SNPs are represented by the bolded alleles. The ribonucleotide residue is preceded by a lower case 'r', followed by the mismatched nucleotide five bases downstream; the C3 spacer is indicated at the 3’ end of the primers

**Fig. 2:**
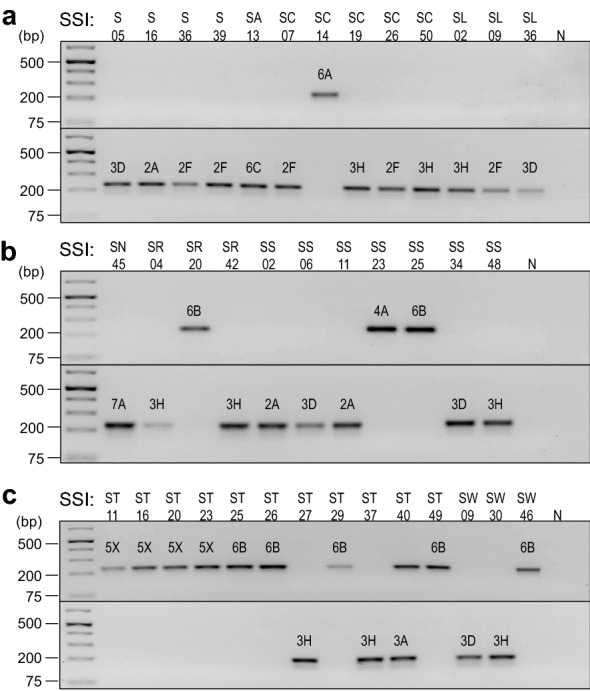
Testing of the specificity of the rhPCR assay against single-spore isolates of *Plasmodiophora brassicae*. Amplification products were resolved by electrophoresis on 1% (w/v) agarose gels prepared with Tris-acetate-EDTA buffer and SYBR Safe gel stain. A GeneRuler 1 kb Plus DNA Ladder (Thermo Scientific, Waltham, MA, USA) was included in the leftmost lane as the marker. The primer pairs rh1-43812R (upper panels) and rh1-43812A (bottom panels) were evaluated against single-spore isolates; **a** S05, S16, S36, S39, SA13, SC07, SC14, SC19, SC26, SC50, SL02, SL09, SL36; **b** SN45, SR04, SR20, SR42, SS02, SS06, SS11, SS23, SS25, SS34, SS48; **c** ST11, ST16, ST20, ST23, ST25, ST26, ST27, ST29, ST37, ST40, ST49, SW09, SW30, and SW46. The corresponding pathotype designation of each isolate is indicated above the respective amplicon. The negative control is displayed in the rightmost lane, as represented by the N

Amplification of *P. brassicae* pathotype single-spore isolates (SSIs) from Table 2 with our developed rhPCR primer pairs generated strong bands of the expected 230 base pair size when using 10 ng of purified genomic DNA template (Fig. 2). The primer pair rh1-43812R was specific to pathotypes of the reference cluster. Amplification of all 13 SSIs from the reference cluster using the reference primer pair produced single bands and yielded no visible PCR products with the alternate primer pair. In contrast, the primer pair rh1-43812A was specific to pathotypes of the alternate cluster. Amplification of all 25 SSIs from the alternate cluster using the alternate primer pair produced bands, while no visible PCR products were obtained with the reference primer pair. The only exception was with the alternate cluster SSI ST40 classified as pathotype 3A, which produced bands of equal intensity with both primer pairs (Fig. 2c).

**Table 2:** Single-spore isolates of *Plasmodiophora brassicae* used during rhPCR assay optimization

Reference 5X cluster isolates	SC14 (6A); SR20 (6B); SS23 (4A); SS25 (6B); ST11 (5X); ST16 (5X); ST20 (5X); ST23 (5X); ST25 (6B); ST26 (6B); ST29 (6B); ST49 (6B); SW46 (6B)
Alternate 3H cluster isolates	S05 (3D); S16 (2A); S36 (2F); S39 (2F); SA13 (6C); SC07 (2F); SC19 (3H); SC26 (2F); SC50 (3H); SL02 (3H); SL09 (2F); SL36 (3D); SN45 (7A); SR04 (3H); SR42 (3H); SS02 (2A); SS06 (3D); SS11 (2A); SS34 (3D); SS48 (3H); ST27 (3H); ST37 (3H); ST40 (3A); SW09 (3D); SW30 (3H);

Each single-spore isolate obtained is given a molecular identification for its isolate name. Canadian Clubroot Differential pathotype designations [2, 4] are indicated in parentheses after each isolate name

**Fig. 3:**
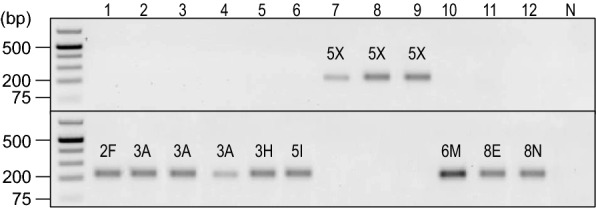
Testing of the specificity of the rhPCR assay against DNA extracted from clubroot galls resulting from infection by *Plasmodiophora brassicae* isolates representing different pathotypes. Amplification products were resolved by electrophoresis on 1% (w/v) agarose gels prepared with Tris-acetate-EDTA buffer and SYBR Safe gel stain. A GeneRuler 1 kb Plus DNA Ladder (Thermo Scientific, Waltham, MA, USA) was included in the leftmost lane as the marker. The primer pairs rh1-43812R (upper panel) and rh1-43812A (bottom panel) were evaluated against *P. brassicae* isolates SACAN-ss3, F3-14, F185-14, F189-14, SACAN-ss1, ORCA-ss3, LG-1, LG-2, LG-3, AbotJE-ss1, F187-14, and CDCN-ss1 (lanes 1–12). The corresponding pathotype of each isolate is indicated above the respective amplicon. The negative control is displayed in the rightmost lane, as represented by the N

The same 230 base pair amplicons were observed when the two primer pairs were tested against DNA extracted from 12 root galls (Table 3) to evaluate the specificity of the rhPCR assay against additional samples, and each sample only amplified with one primer pair (Fig. 3). The sensitivity of the rhPCR assay with DNA extracted from the galls matched that of the DNA from the original SSIs, as the bands were of comparable intensity. Isolates of pathotypes 2F (SACAN-ss3), 3A (F3-14, F185-14, F189-14) and 3H (SACAN-ss1) amplified with the predicted alternate primer pair, and isolates of pathotype 5X (LG-1, LG-2, LG-3) amplified with the predicted reference primer pair. Clustering of pathotypes 5I (ORCA-ss3), 6M (AbotJE-ss1), 8E (F187-14) and 8N (CDCN-ss1) was carried out only after the generation of these results, since these pathotypes were not part of the original SSI collection and we did not have their corresponding sequencing reads. Based on these results, isolates ORCA-ss3, AbotJE-ss1, F187-14 and CDCN-ss1 belong to the alternate cluster.

**Table 3:** Field and single-spore isolates of *Plasmodiophora brassicae* used to evaluate the SNaPshot and rhPCR assays

Isolate identification	Purity^a^	Pathotype^b^
SACAN-ss3	SSI	2F
F3-14	FI	3A
F185-14	FI	3A
F189-14	FI	3A
SACAN-ss1	SSI	3H
ORCA-ss3	SSI	5I
LG-1	FI	5X
LG-2	FI	5X
LG-3	FI	5X
AbotJE-ss1	SSI	6M
F187-14	FI	8E
CDCN-ss1	SSI	8N

^a^Samples were either field isolates (FI) or had been previously purified as single-spore isolates (SSI) [2, 19]

^b^Pathotype designations are based on the Canadian Clubroot Differential set [2, 4]

### SNaPshot

**Fig. 4:**
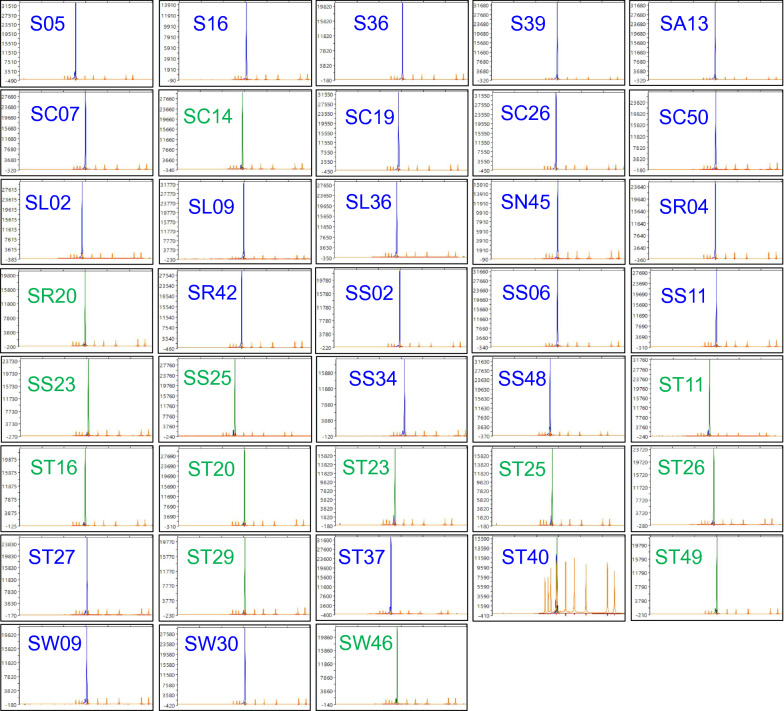
The differentiating capacity of the SNaPshot assay as displayed by capillary electrophoresis of the single-spore isolates of *Plasmodiophora brassicae* listed in Table 2. The SNaPshot primer snpsht1-43,778 was designed against a discriminatory SNP containing an allele of A for the 5X pathotype cluster and an allele of G for the 3H pathotype cluster. Allele A fluoresces green, whereas allele G fluoresces blue. Sequencing results were visualized with Peak Scanner v1.0 (Applied Biosystems, Waltham, MA, USA). The strength of the fluorescence peak is measured against the vertical axis. The size of the SNaPshot product is measured along the x-axis against the GeneScan 120 LIZ size standards (Applied Biosystems, Waltham, MA, USA)

We designed a SNaPshot primer, snpsht1-43,778, to identify the clustering polymorphic allele in the discriminative SNP position. The SNaPshot primer correctly produced fluorescence peaks of the expected color for all 38 SSIs, with green corresponding to the reference cluster and blue corresponding to the alternate cluster (Fig. 4). The SSI ST40, classified as pathotype 3A, yielded both green and blue peaks, showing the existence of both alleles (A and G) in the targeted SNP position. This result is consistent with the results of the rhPCR assay, where amplification of ST40 occurred with both primer pairs, and suggests that this was due to an issue with isolate purity rather than to an error of primer specificity.

**Fig. 5:**
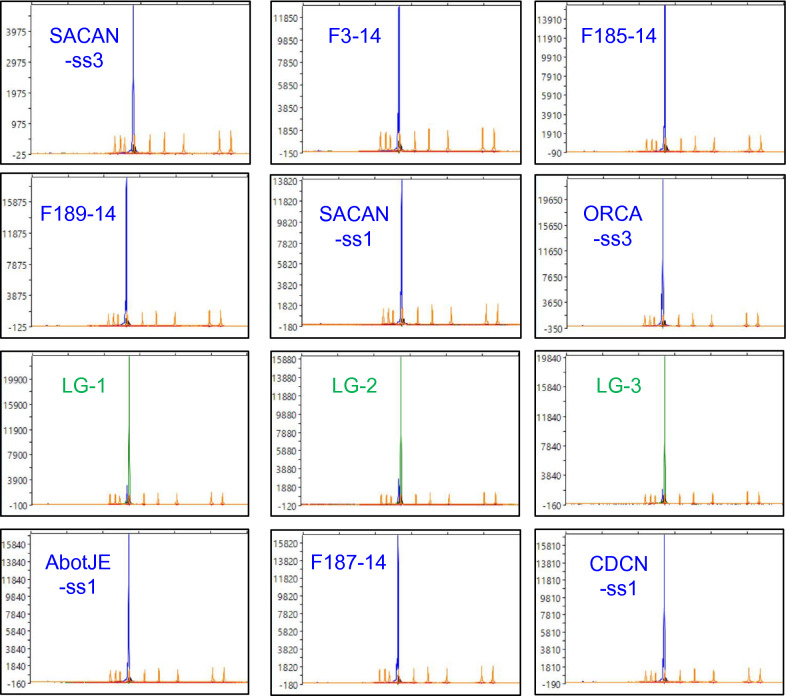
The differentiating capacity of the SNaPshot assay displayed by capillary electrophoresis of DNA extracted from isolates of *P. brassicae* from pathotyped galls. The SNaPshot primer snpsht1-43,778 was designed against a discriminatory SNP containing an allele of A for the 5X pathotype cluster and an allele of G for the 3H pathotype cluster. Allele A fluoresces green, whereas allele G fluoresces blue. Sequencing results were visualized with Peak Scanner v1.0 (Applied Biosystems, Waltham, MA, USA). The strength of the fluorescence peak is measured against the vertical axis. The size of the SNaPshot product is measured along the x-axis against the GeneScan 120 LIZ size standards (Applied Biosystems, Waltham, MA, USA)

The SNaPshot clustering of the DNA samples extracted from canola root galls was consistent with the results of the rhPCR testing (Fig. 5). Isolates classified as pathotypes 2F (SACAN-ss3), 3A (F3-14, F185-14, F189-14) and 3H (SACAN-ss1) belonging to the alternate cluster produced blue fluorescence peaks, and isolates of pathotype 5X (LG-1, LG-2, LG-3) belonging to the reference cluster produced green fluorescence peaks. Pathotypes 5I (ORCA-ss3), 6M (AbotJE-ss1), 8E (F187-14) and 8N (CDCN-ss1) were identified as part of the alternate cluster due to their blue fluorescence. This confirmed that the differentiating SNPs selected for assay development occurred beyond our SSI collection. Furthermore, the sensitivity of the SNaPshot primer with the galled root collections matched that of the DNA from the SSIs as fluorescence peaks were of comparable strengths.

**Fig. 6:**
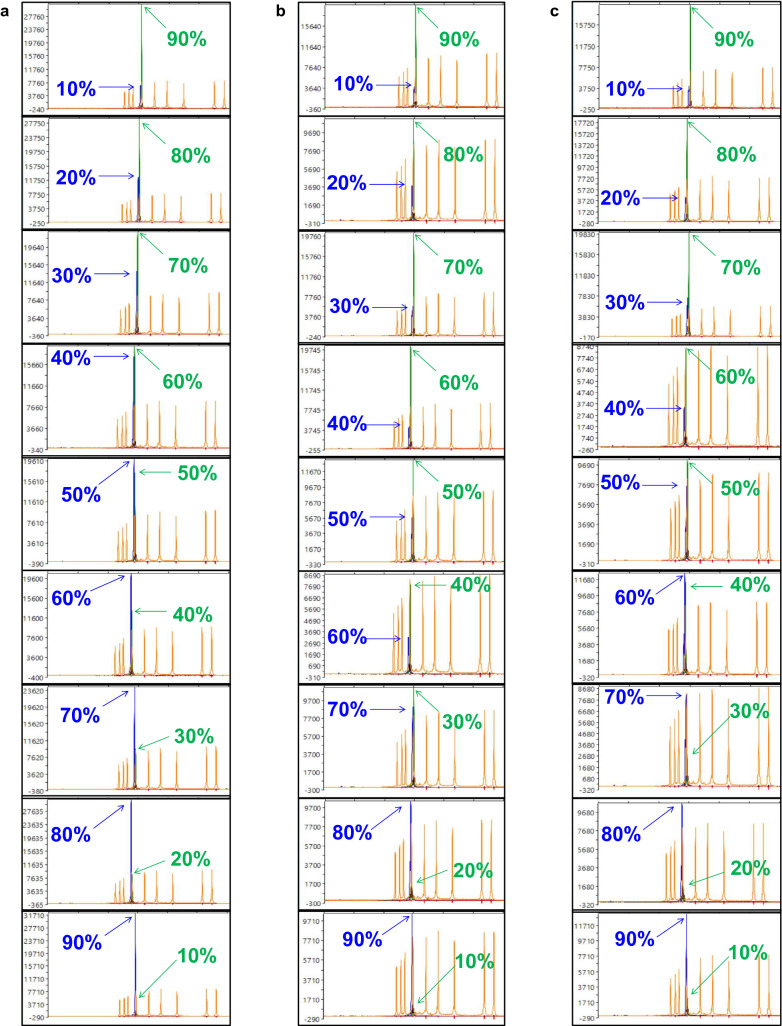
The capacity of the SNaPshot assay to determine the relative abundance of mixtures of *Plasmodiophora brassicae* isolates displayed by capillary electrophoresis. The results shown were obtained with the SNaPshot primer snpsht1-43,778. Three two-isolate mixtures were evaluated at ratios of 10:90, 20:80, 30:70, 40:60, 50:50, 60:40, 70:30, 80:20, and 90:10. The graphs highlight the relative proportion of each allele, an allele of G for the 3H cluster or an allele of A for the 5X cluster. Allele G fluoresces blue, whereas allele A fluoresces green. **a** The first mixture included single-spore isolates SS48 and ST20 from our original collection; **b** the second mixture included the field isolates F3-14 and LG-1 extracted from root galls; and **c** the third mixture included the single-spore isolate SACAN-ss1 and the field isolate LG-2. Sequencing results were visualized with Peak Scanner v1.0 (Applied Biosystems, Waltham, MA, USA). The strength of the fluorescence peak is measured against the vertical axis. The size of the SNaPshot product is measured along the x-axis against the GeneScan 120 LIZ size standards (Applied Biosystems, Waltham, MA, USA)

The capacity of the SNaPshot assay to determine the relative abundance of different isolates was assessed with three two-isolate mixtures (Fig. 6). The first mixture consisted of isolates from our original SSI collection (Fig. 6a), and the second (Fig. 6b) and third mixtures (Fig. 6c) consisted of DNA extracted from root galls. The assay was able to detect a 10% relative allelic proportion in a 10:90 template mixture. However, relative peak strengths were not always proportional to the abundance ratio of the two isolates within each mixture, and therefore this does not represent a quantitative assay.

### Blind testing

**Fig. 7:**
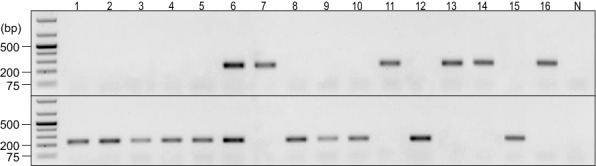
Results of a single-blind evaluation of *Plasmodiophora brassicae* field and single-spore isolates with the rhPCR assay. Amplification products were resolved by electrophoresis on a 1% (w/v) agarose gel prepared with Tris–acetate-EDTA buffer and SYBR Safe gel stain. A GeneRuler 1 kb Plus DNA Ladder (Thermo Scientific, Waltham, MA, USA) was included in the leftmost lane as the marker. The primer pairs rh1-43812R (upper panel) and rh1-43812A (bottom panel) were used to cluster the 16 blinded galls. Samples were disclosed to be isolates SACAN-ss3, ORCA-ss4, AbotJE-ss1, CDCN-ss3, ST27, ST40, LG-2, F3-14, F1-14, CDCN #4-14, SW46, F185-14, SS23, LG-3, SACAN-03-1, and SC14 (lanes 1–16). The negative control is displayed in the rightmost lane, as represented by the N

**Fig. 8:**
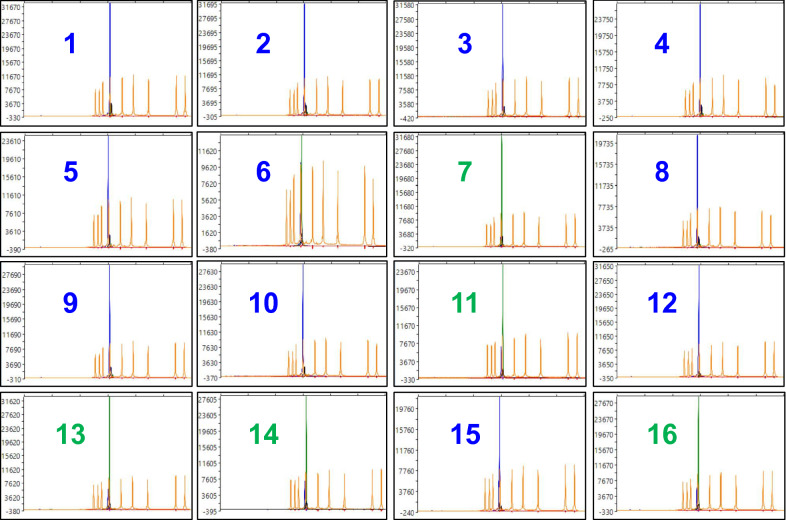
Results of the single-blind study with the SNaPshot assay displayed with capillary electrophoresis. The SNaPshot primer snpsht1-43,778 was used to cluster the 16 blinded galls. The graphs highlight the discriminatory SNP, either an allele of A for the 5X cluster or an allele of G for the 3H cluster. Allele A fluoresces green, whereas allele G fluoresces blue. Samples were disclosed to be isolates SACAN-ss3, ORCA-ss4, AbotJE-ss1, CDCN-ss3, ST27, ST40, LG-2, F3-14, F1-14, CDCN #4-14, SW46, F185-14, SS23, LG-3, SACAN-03-1, and SC14. The sequencing results were visualized with Peak Scanner v1.0 (Applied Biosystems, Waltham, MA, USA). The size of the SNaPshot product is measured along the x-axis against the GeneScan 120 LIZ size standards (Applied Biosystems, Waltham, MA, USA)

The rhPCR and SNaPshot assays were validated in a single-blind study with 16 blinded samples (Table 4). Samples were assigned into either the reference or alternate clusters based on the results of the rhPCR amplification and SNaPshot fluorescence peaks. The rhPCR primer pairs produced the expected 230 base pair amplicon, and band intensity was comparable with earlier testing (Fig. 7). The SNaPshot primer produced either green or blue fluorescence peaks of comparable strength (Fig. 8). After completing the assays, samples 5, 6, 11, 13, and 16 were revealed to be the same SSIs as in our original collection (Table 2), while samples 1, 3, 7, 8, 12, and 14 were revealed to be the same isolates we had previously used for DNA extraction from root galls (Table 4). Sample 6, which was revealed as SSI ST40 classified as pathotype 3A, again produced amplicons with both primer pairs in the rhPCR assay and both blue and green peaks with the SNaPshot assay.

**Table 4:** Identification of blinded root galls infected by *Plasmodiophora brassicae* used in the single-blind study

Sample #	Isolate identification	Purity^a^	Pathotype^b^
1	SACAN-ss3^c^	SSI	2F
2	ORCA-ss4	SSI	5I
3	AbotJE-ss1^c^	SSI	6 M
4	CDCN-ss3	SSI	8 N
5	ST27^d^	SSI	3H
6	ST40^d^	SSI	3A
7	LG-2^c^	FI	5X
8	F3-14^c^	FI	3A
9	F1-14	FI	3D
10	CDCN # 4-14	FI	3H
11	SW46^d^	SSI	6B
12	F185-14^c^	FI	3A
13	SS23^d^	SSI	4A
14	LG-3^c^	FI	5X
15	SACAN-03-1	FI	3H
16	SC14^d^	SSI	6A

While samples had been previously pathotyped, the experiment was conducted without knowledge of pathotype designations until assays were complete

^a^Samples were either field isolates (FI) or had been previously purified as single-spore isolates (SSI)[2, 19]

^b^Pathotype designations are based on the Canadian Clubroot Differential set[2, 4]

^c^Isolates used in earlier testing of DNA extracted from pathotyped galls (Table 3)

^d^SSIs used during the optimization stage of the rhPCR and SNaPshot assays (Table 2)

## Discussion

The aim of this study was to develop *P. brassicae* pathotyping assays for clubroot diagnostics using discriminating polymorphic regions that differentiate pathotype clusters. Molecular pathotyping of *P. brassicae* has been limited up to this point, as only a few assays and molecular markers have been reported. The rhPCR and the SNaPshot assays developed in this study are much faster than the use of the CCD or any other host differential set, generating same day results once DNA is extracted. The technologies behind these two assays show strong potential to be specific and reliable for molecular pathotyping. The SNPs used as molecular markers for the development of the assays were tested and confirmed to be specific to the pathotype clusters from which they were designed. Isolate origin had no effect, since all of the SSIs in our original collection (Table 2) and the DNA extracted from the root galls (Table 3) resulted in the same level of specificity with both the rhPCR and SNaPshot assays, and yielded the same 230 base pair amplicon with the rhPCR assay. This suggests that the polymorphic region selected here is consistent among all isolates.

Unlike a previously reported rhPCR assay [17], the assay reported here was developed using a collection of *P. brassicae* isolates that had been pathotyped on the CCD set. This allows for a more distinct and potentially relevant clustering of pathotypes from Canada, with the ability to link this clustering to the virulence phenotypes of the pathotypes on the hosts of the CCD. The two rhPCR primer pairs simultaneously and specifically amplified the expected pathotype clusters and produced no amplification of pathotypes of the opposing cluster, demonstrating their high specificity for the SNPs in the selected polymorphic region.

The SNaPshot assay is the first of its kind in clubroot diagnostics, as no single base extension assay for the purpose of *P. brassicae* pathotyping has been reported. Template generation with the conventional PCR primer pair produced an amplicon suitable for the extension reaction. The primer sites were conserved among all the SSIs in our original collection and across the pathotyped galls, with the primers consistently producing the expected 305 base pair amplicon that is used as template for the SNaPshot reaction (see Methods). Our selected differentiating SNP and the target site of the SNaPshot primer was adequately situated within the amplicon, as indicated by the successful extension of the SNP. The SNaPshot primer accurately produced green fluorescence peaks for pathotypes of the reference cluster and blue fluorescence peaks for pathotypes of the alternate cluster. The assay also was shown to be sufficiently sensitive to detect both pathotypes in two-isolate mixtures in the relative abundance testing.

The rhPCR and SNaPshot assays were able to differentiate pathotypes of the reference 5X cluster from pathotypes of the alternate 3H cluster; however, the one exception was the SSI ST40 classified as pathotype 3A. With this isolate, amplification of products of comparable intensity was observed with both the reference and alternate rhPCR primer pairs (Fig. 2c), and extension of the SNaPshot primer produced both blue and green peaks (Fig. 4). These mixed results from the SSI ST40 were further confirmed in the single-blind study, where ST40 was revealed to be blinded sample 6 (Table 4), for which amplification occurred with both rhPCR primer pairs (Fig. 7), and both blue and green peaks appeared with the SNaPshot primer (Fig. 8). This indicates that allelic variants of both pathotype clusters are present in the template. In the report where ST40 was first described, it was indicated that while this SSI most closely resembled pathotype 3A, it also displayed characteristics similar to pathotypes 3H, 5X and 6B [2]. As such, the authors of the original study decided to eliminate SSI ST40 from further testing. Since SSI ST40 was supposedly produced from a single-spore, its heterogeneity could reflect an error in the initial single-spore isolation process (e.g., two resting spores attached together), or perhaps mixing during propagation under greenhouse conditions. It will be important to confirm the purity of isolates prior to subjecting a sample for testing during future assay development, to enable the accurate interpretation of results and to prevent false positives or negatives.

The accuracy and sensitivity of the rhPCR and SNaPshot assays should facilitate the analysis of *P. brassicae* field populations for the presence of heterogeneity. For example, the field isolates (FIs) LG-1, LG-2, and LG-3, all of which were classified as pathotype 5X [1, 4], presented miniscule but notable blue peaks in addition to the expected green peaks with the SNaPshot assay (Fig. 5). This indicates the presence of another pathotype of the 3H cluster (in much smaller proportions) within the 5X FIs, likely reflecting the coexistence of multiple pathotypes in one field gall [2, 19, 20]. The virulence patterns of FIs on differential hosts often reflect the ‘predominant’ pathotype found in a root gall, and may not capture the extent of heterogeneity in *P. brassicae* populations from the field [19, 21]. A recent study investigating the purity of pathogen populations collected from field galls confirmed this to be the case, with most representing a mixture of pathotypes and showing some heterogeneity [20].

The production of reproducible and accurate data relies upon the DNA extraction and purification methods used. In this study, DNA was collected by means of CTAB extraction [22] followed by phenol-chloroform liquid-liquid purification for all of the SSIs and FIs evaluated, as these methods have been shown to produce high quality DNA for downstream PCR applications. The quality and quantity of the purified DNA were verified on a NanoDrop spectrophotometer (Thermo Scientific, Waltham, MA, USA). We have not tested the assays against DNA extracted using different methodologies, and therefore, isolates prepared with other extraction methods must be tested to ensure rhPCR amplification and SNaPshot sequencing remain consistent. PCR and sequencing is affected by low yields, low integrity, and impurities in the presence of contaminants and inhibitors [23, 24]. Since high quality and quantity of template DNA is critical for PCR-based and sequencing studies, appropriate DNA extraction and purification procedures are essential for consistency during both assay optimization and testing. In addition, the primers developed here were designed to identify *P. brassicae* pathotypes present in root galls, and have not been tested for the detection of pathotypes in soil or water samples. The assays should be suitable for evaluation of these types of samples if comparable DNA yield, integrity and quality are obtained during sample preparation.

Initially, the intention of this study was to develop assays to distinguish pathotype 5X from pathotype 3H. However, we found that the discriminatory polymorphic region we selected for our analysis could group many other pathotypes into one of these two main clusters, as listed in Table 2. It is possible that the two pathotype clusters observed in this study correspond to the two genetically distinct populations of *P. brassicae* identified in an earlier reported study [14], with the 5X and 3H clusters correlating with their “virulent” and “avirulent” populations, respectively. Additional testing will be necessary to confirm this hypothesis.

During the initial primer design stage of this study, we were limited to the whole-genome SNP profiles of the 38 SSIs in our collection. Additional pathotypes for which we did not have sequencing reads were only later classified into the clusters, based on the results of the rhPCR and SNaPshot assays. Specifically, the isolates ORCA-ss3, AbotJE-ss1, F187-14 and CDCN-ss1, corresponding to pathotypes 5I, 6 M, 8E and 8 N, respectively, were tested without prior knowledge of which cluster they grouped with, as they were not originally considered nor did we have their corresponding whole-genome sequences. This consideration would also apply to any new *P. brassicae* pathotypes identified and tested in the future, as the primers were not initially designed to target their variants. If based on these two assays exclusively, clustering of new pathotypes would depend on the allelic variant in the discriminatory SNP positions, which might or might not be consistent with their CCD designation(s) based on virulence phenotype(s). Moreover, the longevity and stability of the region targeted by these particular rhPCR and SNaPshot primers are not known, since genome rearrangements or shifts in the pathogen population could occur in response to selection pressure. These polymorphic regions have not undergone further testing in other studies, and were first selected here based on the genomic DNA sequencing of our isolates. Similarly, mutations in the discriminating SNPs could affect the effectiveness of the assays [25, 26].

The rhPCR technology has the capacity to be optimized into a multiplex reaction for the simultaneous detection of multiple targets. A different primer pair for each target is required and amplicons must be of different lengths, as multiplexing relies on electrophoretic separation of bands [27]. The target is identified based on the molecular weight of the electrophoretic band, and pathotype clustering is determined based on the presence or absence of that band. Since rhPCR is a PCR-based approach, the capacity to adapt rhPCR primers into a quantitative assay is an advantage of this technique [28]; primers and rhPCR components can be incorporated in a dye-based or probe-based PCR. For a dye-based qPCR (SYBR-green), the mix of rhPCR components and dye is sufficient. For a probe-based qPCR, an additional polymorphic region for the probe is needed between the primers, or one of the polymorphic regions of one of the primers would have to be used as probe-binding region, displacing the position of one of the primers. The application of qPCR also provides greater sensitivity for detection of low frequency DNA, since the initial amount of target DNA is directly correlated with an early or late exponential curve of amplification [29, 30]. A multiplex quantitative rhPCR assay would require the design of additional primer pairs and labeling of probes with distinct fluorophores for each amplicon.

The main advantage of the SNaPshot technology is its capacity to detect up to four alleles per targeted site by means of fluorescent ddNTPs variants. It would therefore be ideal if a SNaPshot primer is designed against a polymorphic SNP that distinguishes four distinct pathotype clusters (although this level of polymorphism is unlikely for a single site). In addition, SNaPshot is scalable through a multiplex reaction, where discriminatory SNPs from several different genomic regions can be examined concurrently. This would facilitate efficient and rapid testing. Differential primer lengths for each targeted SNP are required, however, since the product size of the fluorescence peak is determined by the length of the primer. Product size is measured along the x-axis, and therefore, the size of the product determines the targeted SNP and the peak color determines the allele in that corresponding SNP.

The rhPCR and SNaPshot assays in this study can only distinguish pathotypes of the 5X cluster from the 3H cluster, since the rhPCR primer pairs target only one set of allelic variants and the SNaPshot primer targets one SNP. To be able to distinguish isolates within the clusters further (ideally down to their individual CCD pathotype designations), multiple primers targeting various differential SNPs would need to be designed and multiple reactions would have to be carried out in parallel or multiplexed. In this case, the development of a multiplex reaction would increase efficiency. The sequencing reads of the SSIs in this study were assembled against the 2015 e3 reference genome [31]. We are currently re-aligning the SSI sequencing reads against the 2019 re-assembled e3 reference genome [32]. The 2019 genome is more accurate and reliable than its 2015 counterpart, containing an improved genome assembly with longer continuous sequences. Moving forward, we will be using the re-aligned whole-genome SNP profiles from our isolates for assay development.

## Conclusions

This study describes the development of two independent rapid and sensitive technologies for *P. brassicae* pathotyping, an rhPCR and a SNaPshot assay. The high-throughput potential and accuracy of both assays makes them promising as SNP-based pathotype identification tools for routine testing of *P. brassicae* pathotypes. The rhPCR technology is a highly sensitive approach that can be optimized into a quantitative assay, using widely available lab equipment, while the main advantage of SNaPshot is its ability to multiplex samples and alleles in a single reaction. To our knowledge, this is the first report of an rhPCR assay for the detection of *P. brassicae* pathotype clusters as classified by the CCD set, and the first single-base extension assay for the purpose of *P. brassicae* pathotyping.

## Methods

### SNP selection

Thirty-eight *P. brassicae* single-spore isolates were included in this study. The isolation of purified genomic DNA from resting spores used in our study was previously reported [33]. The DNA was quantified with a Qubit 2.0 DNA HS Assay (Thermo Scientific, Waltham, MA, USA) and DNA quality was assessed with a TapeStation Genomic DNA Assay (Agilent Technologies, Santa Clara, CA, USA). Samples were then sent to Admera Health (South Plainfield, NJ, USA) for library preparation, next-generation sequencing, and variant calling. The sequencing library was prepared using a KAPA Hyper Prep Kit (Roche, Basel, Switzerland) as per the manufacturer’s recommendations. Library quality and quantity were evaluated with a Qubit 2.0 DNA HS Assay (Thermo Scientific, Waltham, MA, USA) and TapeStation High Sensitivity D1000 Assay (Agilent Technologies, Santa Clara, CA, USA). The prepared library was then sequenced (2 × 150 bp reads) on an Illumina® HiSeq X instrument. Sequencing reads were aligned to the 2015 e3 reference genome for *P. brassicae* [31] (European Nucleotide Archive project PRJEB8376). Variants were called from high quality aligned reads using HaploTypeCaller [34–37] with filters of overall read depth equal to or larger than 15 (DP ≥ 15) and quality equal to or larger than 40 (GQ ≥ 40) to produce variant call format (vcf) files per each isolate. SOAPdenovo v2.01 [38, 39] was used to assemble the reads into draft assemblies.

**Fig. 9:**
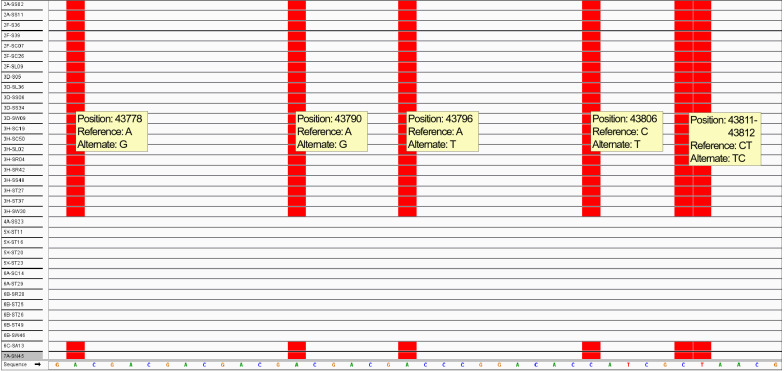
Example of the alignment of *Plasmodiophora brassicae* isolates from this study with the *P. brassicae* e3 reference genome [31] using the Integrated Genomics Viewer [40]. Each row represents an individual single-spore isolate, and the corresponding isolate name is displayed in the left vertical axis. The sequence of the reference genome is shown along the bottom horizontal axis. This presented region belongs to genome coordinates 43,777 to 43,816 of contig 1, and provides a clear genomic differentiation between the 5X pathotype cluster and the 3H cluster. The red boxes indicate a SNP against the reference genome, and the allelic variant in the SNP position is referred to the “Alternate” allele. Isolates of pathotype 3H belong to the alternate cluster according to this particular polymorphic region. Therefore, the other isolates containing the alternate allelic variants are grouped with pathotype 3H to form the alternate 3H cluster. Likewise, isolates of pathotype 5X belong to the reference cluster. Therefore, the other isolates containing the reference allelic variants are grouped with pathotype 5X to form the reference 5X cluster

We loaded the resulting vcf files into the Integrative Genomics Viewer [40] to visualize polymorphisms and identify candidate SNP loci among the 11 CCD pathotypes represented in our 38 SSIs (Fig. 9). All polymorphisms utilized for our assays came from alignments of all our isolates classified using the CCD set. To confirm the polymorphic region found in contig 1 [31] that differentiates the 5X pathotype cluster from the 3H cluster (Table 2), a conventional PCR primer pair was designed to amplify the region encompassing the SNPs through Sanger sequencing [41]. Forward primer SEQ1-43778fw 5′-GCCTGTCGAACGTCTGTT-3′ and reverse primer SEQ1-43778rv 5′-ATAAAGTCTGGACACGAGAACG-3′ were designed using PrimerQuest (Integrated DNA Technologies, Coralville, IA, USA) with parameters that included primer length ranging between 18–24 bases, GC content ranging between 40–60%, and melting temperature of 60 °C. This set produced a 508 base pair amplicon to confirm SNPs used for both the rhPCR and SNaPshot assays. The primers were evaluated for specificity with command line BLAST v. 2.6.0 against the reference e3 *P. brassicae* genome [31]. The argument—task "blastn-short" was used as this task is optimized for short sequences of less than 30 nucleotides. The primers were also subjected to a BLAST search in the National Center for Biotechnology Information (NCBI) online database (http://blast.ncbi.nlm.nih.gov/Blast.cgi) to ensure specificity to *P. brassicae*.

Three SSIs from each cluster were selected for Sanger sequencing of the amplicons to validate the presence of polymorphisms detected using whole genome sequencing. The SSIs ST11 (5X), ST23 (5X) and SR20 (6B) were tested from the reference 5X cluster, and SSIs SL09 (2F), SS48 (3H), and SW30 (3H) were tested from the alternate 3H cluster [2]. PCR analyses were carried out in a 20 µL final volume containing 1X reaction buffer, 2 mM MgCl_2_, 0.2 mM dNTPs, 0.4 µM of each forward and reverse primers, 1 U Platinum Taq DNA polymerase (Invitrogen, Waltham, Massachusetts, USA), 10 ng of genomic DNA as template, and 13.7 μl nuclease-free water. All reactions were conducted in a Veriti 96-Well Thermal Cycler (Applied Biosystems, Waltham, MA, USA) under the following cycling conditions: 2 min at 94 °C, followed by 40 cycles of 30 s at 94 °C, 1 min at 63 °C, and 1 min at 72 °C, with a final 10 min extension at 72 °C. Samples were held at 4 °C. Four technical replications of each sample were performed. The PCR products from one replicate per each sample were resolved by electrophoresis on a 1% agarose gel to confirm the presence of specific amplification, product size and intensity. The other three replicates were combined and purified using the Wizard SV Gel and PCR Cleanup System (Promega, Madison, WI, USA) following the manufacturer’s specifications. The quality and quantity of purified DNA were verified on a NanoDrop spectrophotometer (Thermo Scientific, Waltham, MA, USA), then sent for Sanger sequencing [41] at the Molecular Biology Service Unit, Department of Biological Sciences, University of Alberta (Edmonton, AB, Canada). The resulting sequences were visualized and SNPs were confirmed with Sequencher 5.0 (Gene Codes Corporation, Ann Arbor, MI, USA).

### RNase H2-dependent PCR

The reference rhPCR primer pair was designed to amplify isolates of the 5X cluster; it was referred as the reference cluster since the SNPs also belonged to the *P. brassicae* e3 reference genome [31]. The alternate rhPCR primer pair was designed to amplify isolates of the 3H cluster (Table 1). In addition to the differentiating SNPs positioned against the ribonucleotide bases, the primers were positioned in a polymorphic region that would allow for multiple SNPs to increase specificity. There were five SNPs between the forward primers and two SNPs between the reverse primers. These sets produced a 230 base pair amplicon. The specificity of the primers was evaluated with command line BLAST v. 2.6.0 against the e3 reference genome [31]. The primers were also subjected to a BLAST search in the NCBI online database to ensure specificity to *P. brassicae*.

The specificity of the primers and the rhPCR block-cleavable technology was evaluated against gBlocks gene fragments (Integrated DNA Technologies, Coralville, IA, USA), double-stranded synthetic oligonucleotides. One gBlock was designed to replicate the 5X polymorphic region sequence, and another was designed to replicate the 3H polymorphic sequence. The gBlock gene fragment contained the 230 base pair rhPCR amplicon in its entirety, plus an additional 100 base pairs upstream and downstream from the amplicon. PCR analyses were carried out in a 20 µL final volume containing 1X reaction buffer (Applied Biosystems, Waltham, MA, USA), 2 mM MgCl_2_, 0.2 mM dNTPs, 0.4 µM of each forward and reverse primer, 1 U Platinum Taq DNA polymerase (Invitrogen, Waltham, MA, USA), 5.2 mU RNase H2 enzyme, and 5 ng gBlock as template. The gBlock testing was run in a Veriti 96-Well Thermal Cycler (Applied Biosystems, Waltham, MA, USA) under the following cycling conditions: 2 min at 94 °C, followed by 12 cycles of 10 s at 94 °C and 30 s at 70 °C. Samples were held at 4 °C until the PCR products were electrophoresed on a 1% agarose gel. The block-cleavable technology was also tested by repeating the PCR, but with the RNase H2 enzyme excluded from the master mix as a control.

The rhPCR primer pairs were then evaluated and optimized against the SSIs in our collection: 13 isolates belonging to the 5X cluster and 25 isolates belonging to the 3H cluster (Table 2). PCR analyses were carried out in a 20 µL final volume containing 1X reaction buffer, 2 mM MgCl_2_, 0.2 mM dNTPs, 0.4 µM of each forward and reverse primer, 1 U Platinum Taq DNA polymerase (Invitrogen, Waltham, Massachusetts, USA), 5.2 mU RNase H2 (Integrated DNA Technologies, Coralville, IA, USA), and 10 ng genomic DNA as template. The reaction was run in a Veriti 96-Well Thermal Cycler (Applied Biosystems, Waltham, MA, USA) under the following cycling conditions: 2 min at 94 °C, followed by 35 cycles of 10 s at 94 °C and 30 s at 70 °C. Annealing temperatures and extension times for PCR were determined according to the primer sequence and amplicon size. Samples were held at 4 °C until the amplicons were electrophoresed on a 1% agarose gel.

### SNaPshot

A conventional PCR primer pair was designed to generate the template for the SNaPshot extension reaction. The primer sites to generate this product were conserved among the 38 SSIs and targeted a region that contained the differentiating SNP. The same forward primer SEQ1-43778fw previously designed for Sanger sequencing was used in conjunction with a newly designed reverse primer SEQ1-43778rv2 5′-CTCGAACTCTTTGTCGTCGTT-3′. This set generated a 304 base pair amplicon corresponding to coordinates 43,671 to 43,974 from contig 1 of the e3 reference genome [31]. The selected differentiating SNP was used earlier as one of the SNPs within the forward primer region of our rhPCR assay. A SNaPshot primer snpsht1-43,778 5′-AAAAAAACGATAACGTCGTGGACGACGGCG-3′ was designed upstream of the polymorphic base to distinguish pathotypes. A seven nucleotide non-homologous polyA tail was added to the 5’ end to bring the length of the primer to 30 nucleotides long, the minimum length recommended for the assay (Applied Biosystems, Waltham, MA, USA). The complementary region between the primer and template was kept at 23 nucleotides, to maintain an annealing temperature of 50 °C that matched the annealing temperature (50 °C) of the SNaPshot control primer (Applied Biosystems, Waltham, MA, USA). The primer was subjected to reverse phase high performance liquid chromatography purification (Integrated DNA Technologies, Coralville, IA, USA).

All of the SSIs listed in Table 2 were also tested in the SNaPshot assay. Template generation was carried out in a 20 µL final volume PCR containing 1X reaction buffer, 2 mM MgCl_2_, 0.2 mM dNTPs, 0.4 µM of each forward and reverse primer, 1 U Platinum Taq DNA polymerase (Invitrogen, Waltham, Massachusetts, USA), and 10 ng genomic DNA as template. The reaction was run in a Veriti 96-Well Thermal Cycler (Applied Biosystems, Waltham, MA, USA) under the following cycling conditions: 2 min at 94 °C, then 40 cycles of 30 s at 94 °C, 1 min at 63 °C, and 1 min at 72 °C, followed by a final 10 min extension at 72 °C. Samples were held at 4 °C. Four technical replications were included for each sample. The PCR products of a single replicate from each sample were electrophoresed on a 1% agarose gel to confirm the presence of the specific amplicon and product intensity. The other three replicates were combined and purified using the Wizard SV Gel and PCR Cleanup System under manufacturer specifications (Promega, Madison, WI, USA). gBlocks corresponding to each 5X and 3H cluster were also designed and used to run control reactions in parallel. 5 ng of gBlocks were used as template instead of 10 ng genomic DNA, to reduce the copy number of this region sequence, and only 12 cycles were conducted in the PCR instead of 40 cycles, as recommended by the manufacturer (Integrated DNA Technologies, Coralville, IA, USA).

The SNaPshot Multiplex Kit (Thermo Scientific, Waltham, MA, USA) was used for the extension reaction in a 10 µL final volume containing 1X master mix (Thermo Scientific, Waltham, MA, USA), 0.2 µM SNaPshot primer, and 0.2 pmol SNaPshot template. The extension reaction was carried out in a Veriti 96-Well Thermal Cycler (Applied Biosystems, Waltham, MA, USA) under the following cycling conditions: 25 cycles of 10 s at 96 °C, 5 s at 50 °C, and 30 s at 60 °C, then held at 4 °C. Control reactions with a control template and control primers supplied by the SNaPshot Multiplex Kit (Thermo Scientific, Waltham, MA, USA) were run in parallel under the same cycling conditions. Extension reaction products were then subjected to a post-extension treatment with SAP (New England BioLabs, Ipswich, MA, USA) to remove any unincorporated ddNTPs. One unit of SAP was added to each sample, and then incubated for 60 min at 37 °C, followed by 15 min at 75 °C, and held at 4 °C.

Treated extension products were then prepared in a 96-well plate for capillary electrophoresis. Each injection was performed at a final volume of 10 μL containing 9 μL Hi-Di formamide (Applied Biosystems, Waltham, MA, USA), 0.5 μL GeneScan 120 LIZ size standards (Applied Biosystems, Waltham, MA, USA), and 0.5 μL extension product. The plate was incubated for 5 min at 95 °C, and capillary electrophoresis was carried out in an ABI Prism 3730 Genetic Analyzer (Applied Biosystems, Waltham, MA, USA) at the Molecular Biology Service Unit, Department of Biological Sciences, University of Alberta (Edmonton, AB, Canada). The Peak Scanner v1.0 (Applied Biosystems, Waltham, MA, USA) was used to determine the SNP allele based on the resulting fluorescence peak.

### Extraction of DNA from root galls for evaluating the rhPCR and SNaPshot assays

The performance of the SNaPshot and rhPCR assays was evaluated with 12 canola root galls representing different field and single-spore isolates that had been previously pathotyped using the CCD set (Table 3). The *P. brassicae* DNA from the galls was isolated using the cetyltrimethylammonium bromide (CTAB) extraction method [22], followed by phenol–chloroform purification. The CTAB lysis buffer was prepared with 2% CTAB (w/v), 100 mM Tris–HCl (pH 8.0), 20 mM EDTA (pH 8.0), and 1.4 mM NaCl, and the pH of the buffer was adjusted to 8.0 prior to sterilization in an autoclave. The galls were frozen at -80 °C for 24 h, and then ground in liquid nitrogen with a mortar and pestle. The resultant ground sample (200 mg from each gall) was transferred into a 2 mL microcentrifuge tube and 600 µL of CTAB extraction buffer was added. The samples were incubated at 60 °C for 20 min, during which samples were mixed by inversion every 5 min. After incubation, an equal volume of 600 µL phenol:chloroform:isoamyl alcohol (25:24:1, v/v) was added, vortexed, and centrifuged at 14,000 rpm for 5 min. The top aqueous phase (supernatant) was transferred to a new 2 mL microcentrifuge tube, and was subjected to two more rounds of phenol:chloroform:isoamyl alcohol DNA purification. The purified DNA was then precipitated in 700 µL of 100% ice-cold isopropanol; samples were mixed by inversion, placed on ice for 10 min, and then centrifuged at 14,000 rpm for 8 min. The isopropanol was discarded and the precipitated DNA pellet was washed with 500 µL of 80% ice-cold ethanol; the sample was vortexed until the pellet detached off the tube, and then centrifuged at 14,000 rpm for 3 min. The ethanol was discarded and the remaining pellet was left at room temperature to air dry. Once dried, the DNA was dissolved and resuspended in 100 µL sterile nuclease-free water. The concentration and purity of each sample were determined with a NanoDrop 1000 spectrophotometer (Thermo Scientific, Waltham, MA, USA), and DNA integrity was assessed by loading 200 ng per sample onto a 1% agarose gel. Samples were then diluted to a working concentration of 5 ng µL^−1^ with sterile nuclease-free water and stored at −20 °C. The samples were tested in the SNaPshot and rhPCR assays under the same conditions as described above.

### Testing of relative abundance

Different proportions of mixed isolates were tested to assess the capacity of the SNaPshot assay to determine relative abundances. Three different two-isolate mixtures were evaluated with the different proportions of 10:90, 20:80, 30:70, 40:60, and 50:50 (Table 5). Mixtures were prepared prior to template generation to simulate conditions where a root gall developed from a mixed infection by more than one pathotype. Ten ng of total genomic DNA was used for the PCR. The entire SNaPshot assay procedure from template generation to capillary electrophoresis followed the same protocol as described earlier.

**Table 5:** *Plasmodiophora brassicae* isolate mixtures generated to assess the capacity of the SNaPshot assay to determine relative abundances

Isolates^a^	Proportions	Genomic DNA (ng)^b^
SS48(3H): ST20(5X)	10: 90	1: 9
	20: 80	2: 8
	30: 70	3: 7
	40: 60	4: 6
	50: 50	5: 5
	60: 40	6: 4
	70: 30	7: 3
	80: 20	8: 2
	90: 10	9: 1
F3-14(3A): LG-1(5X)	10: 90	1: 9
	20: 80	2: 8
	30: 70	3: 7
	40: 60	4: 6
	50: 50	5: 5
	60: 40	6: 4
	70: 30	7: 3
	80: 20	8: 2
	90: 10	9: 1
SACAN-ss1(3H): LG-2(5X)	10: 90	1: 9
	20: 80	2: 8
	30: 70	3: 7
	40: 60	4: 6
	50: 50	5: 5
	60: 40	6: 4
	70: 30	7: 3
	80: 20	8: 2
	90: 10	9: 1

^a^SS48, ST20, and SACAN-ss1 were single-spore isolates; F3-14, LG-1 and LG-2 were field isolates; pathotype designations based on the Canadian Clubroot Differential set [2, 4] are indicated in parentheses

^b^10 ng total genomic for template generation with primers SEQ1-43778fw and SEQ1-43778rv2

### Blind testing

Blind testing was conducted with the rhPCR and SNaPshot assays. While the isolates corresponding to the galls had been previously pathotyped, the experiment was conducted without knowledge of pathotype designations in a single-blind experiment. *P. brassicae* DNA from 16 blinded galls was extracted according to the CTAB method [22] following the same procedure as described earlier. Blinded samples were subjected to both rhPCR and SNaPshot assays under the conditions described above.

## Data Availability

The datasets used or generated in this study are included in the published article or are available from the corresponding author on reasonable request.

## Abbreviations

CR: Clubroot-resistant
CCD: Canadian clubroot differential
rhPCR: RNase H2-dependent PCR
SSI: Single-spore isolate
FI: Field isolate
vcf: Variant call format
SNP: Single nucleotide polymorphism
BLAST: Basic local alignment search tool
ddNTP: Dideoxynucleotide
